# The mental health of young doctors during the omicron wave

**DOI:** 10.1192/j.eurpsy.2023.1241

**Published:** 2023-07-19

**Authors:** M. A. Ghrab, I. Sellami, A. Hrairi, M. Hajjaji, K. Jmal Hammami, M. L. Masmoudi

**Affiliations:** 1 Department of occupational medicine, University Hospital Hedi Chaker, Sfax, Tunisia; 2 University of Sfax, Sfax, Tunisia

## Abstract

**Introduction:**

The healthcare environment is a special work environment. It exposes the staff to physical and psychological constraints. Interns starting their careers during this pandemic were exposed to additional stressors in the time of the COVID-19 pandemic.

**Objectives:**

We aimed to screen for depression and generalized anxiety disorder among medical interns during the COVID-19 pandemic.

**Methods:**

We conducted a cross-sectional study, through a pre-established anonymous questionnaire for Tunisian medical interns. This questionnaire was shared online on social networks in April 2022. Patient Health Questionnaire-9 (PHQ-9) and General Anxiety Disorder-7 (GAD-7) were used. The collected data were processed and analyzed by IBM SPSS statistics software.

**Results:**

Our population consisted of 82 interns. The average age was 25.47±1.84 and 76.8% were female. Sixty-one per cent of our population had priors of COVID-19 infection. The mean of the PHQ-9 score was 10.90±6.34. Only 12.2% had a normal PHQ-9 score and nearly one-third of interns had self-harm and suicidal thoughts. Symptoms of moderate to severe depression were observed in 25.6% of interns. The mean of the GAD-7 score was 9.17±5.20. A further specialized evaluation was required in 39.1% of interns who scored 10 or greater. Bivariate analysis showed that the PHQ-9 and GAD-7 scores were correlated (p=0.002, r= 0.78). They were not associated with sex, age, or medical history.

**Conclusions:**

Medical interns, who just started their professional careers during an important COVID-19 wave, suffered greatly in terms of mental health. Serious attention and evaluation are needed for this fragile young category of healthcare professionals.

**Disclosure of Interest:**

None Declared

